# Incidence and Prognostic Role of Pleural Effusion in Patients with Pulmonary Embolism: A Systematic Review and Meta-Analysis

**DOI:** 10.3390/jcm12062315

**Published:** 2023-03-16

**Authors:** Ping Li, Jing An, Shuyan Wang, Xueru Hu, Tingting Zeng, Chun Wan, Yongchun Shen, Tao Wang

**Affiliations:** Department of Respiratory and Critical Care Medicine, West China Hospital of Sichuan University and Division of Pulmonary Diseases, State Key Laboratory of Biotherapy of China, Chengdu 610041, China

**Keywords:** pulmonary embolism, pleural effusion, systematic review, meta-analysis

## Abstract

Background: Pleural effusion is a common pulmonary embolism (PE) complication, which has been documented to increase the risk of death in PE and relate to disease progression. However, the incidence of pleural effusion varies among studies and its association with PE outcome is still unclear. This study sought to determine the pooled incidence and prognostic value of pleural effusion events in patients with PE. Methods: We systematically searched the PubMed, EMBASE, SCOPE, Web of Science, Cochrane, LILACS, CINAHL, EBSCO, AMED, and OVID databases from the inception of each database to 7 September 2022 with a restriction on human studies, to identify studies assessing the association between pleural effusion and PE including all prospective and retrospective clinical studies. An exploratory meta-analysis was performed using a random-effects model. We evaluated the heterogeneity and performed subgroup analyses. Results: The final meta-analysis included 29 studies involving 13,430 PE patients. The pooled incidence of pleural effusion in PE patients was 41.2% (95% CI: 35.7–46.6%), which tended to be unilateral (pooled incidence: 60.8%, 95% CI: 45.7–75.8%) and small (pooled incidence: 85.9%, 95% CI: 82.6–89.1%). Pooled analysis using a random-effects model (I^2^ = 53.2%) showed that pleural effusion was associated with an increased risk of 30-day mortality (RR 2.19, 95% CI: 1.53–3.15, *p* < 0.001, I^2^ = 67.1%) and in-hospital mortality (RR 2.39, 95% CI: 1.85–3.09, *p* < 0.001, I^2^ = 37.1%) in patients with PE. Conclusions: Our meta-analysis found that PE patients had a high incidence of pleural effusion, which was usually unilateral and small. Pleural effusion generally increases 30-day and in-hospital mortality in patients with PE, and it is recommended that physicians be aware of the risk of death from PE, especially when patients have pleural effusion. Further investigations focusing on PE with pleural effusion are warranted.

## 1. Introduction

Pulmonary embolism (PE) is a common and fatal medical condition with high morbidity and mortality, characterized by occlusion of the pulmonary arteries [1], which accounts for death in 5–10% of hospitalized patients [2] and is the third-leading cause of death among hospitalized patients in the United States [3]. Epidemiological studies have revealed that the annual incidence rates for PE range from approximately 30 to 115 per 100,000 people and longitudinal investigations have demonstrated an increasing annual incidence of PE with time [4]. PE accounts for approximately 300,000 deaths, ranking among the top causes of death from cardiovascular disease [4]. Although PE mortality has decreased in recent years as a result of progress in PE treatment—from anticoagulation or systemic thrombolysis, to interventions [5,6]—it remains a diagnostic and therapeutic challenge.

Although pleural effusion is frequently observed in PE patients, it is challenging to pinpoint the precise prevalence of pleural effusion in PE patients due to conflicting research results [7]. However, PE, the fourth most common cause of pleural effusion after congestive heart failure, cancer, and pneumonia [8,9], is also the most underdiagnosed condition among pleural effusion patients [10]. Although emerging evidence suggests that pleural effusion has a high incidence among PE patients and is closely associated with the prognosis of PE, the results of recent studies on the effect of pleural effusion on the prognosis of PE patients are inconsistent [11,12]. In clinical practice, the question of whether the occurrence of pleural effusion reflects high-risk PE is often raised, and the mortality rate of high-risk PE is as high as 30% if left untreated [13]. Therefore, timely identification and adequate treatment of high-risk PE are essential. Clearly, it is important to understand the clinical correlation between pleural effusion and mortality in PE patients, and to adequately stratify and guide the treatment of PE patients.

Given how common pleural effusion in PE patients is, it is important to precisely quantify its association with PE patient outcomes. Therefore, we filled this knowledge gap by conducting a systematic review and meta-analysis of all the current articles, including all prospective and retrospective clinical studies, to assess the incidence and prognostic value of pleural effusion on short-term (30-day or in-hospital) mortality in PE patients and to inform future research and practice.

## 2. Materials and Methods

### 2.1. Search Strategy

This systematic review and meta-analysis followed the Meta-analysis of Observational Studies in Epidemiology (MOOSE) (Table S1) [14] and the Preferred Reporting Items for Systematic Reviews and Meta-Analysis (PRISMA) [15] guidelines. We searched PubMed, EMBASE, SCOPE, Web of Science, Cochrane, LILACS, CINAHL, EBSCO, AMED, and OVID databases to assess the association between pleural effusion and PE from the inception of each database to 7 September 2022, restricted to human studies. The search strategy is a combination of keywords and subject headings. The following search terms were applied: ((“Pulmonary Embolism”[Mesh]) OR (“pulmonary embolism”[Title/Abstract])) OR (“pulmonary thromboembolism”[Title/Abstract])) OR (“PTE”[Title/Abstract])) OR (“pulmonary thrombosis”[Title/Abstract])) AND ((“Pleural Effusion”[Mesh]) OR (“pleural effusion”[Title/Abstract])) OR (“pleural fluid”[Title/Abstract])). Furthermore, the references of pertinent studies were also manually reviewed to identify potentially eligible articles.

### 2.2. Study Selection

The inclusion criteria were as follows: (1) all published original studies comprising randomized controlled trials (RCTs), cohort, case-control, or cross-sectional study designs with samples ≥ 20 patients; (2) patients with PE with or without pleural effusion; (3) presenting clear diagnostic or assessment criteria for pleural effusion and PE; and (4) assessing the incidence of pleural effusion in PE and reporting data on short-term mortality, including 30-day or in-hospital mortality for all patients.

The exclusion criteria were as follows: (1) conference abstracts; (2) editor’s comments; (3) duplicated information provided by the same author and coming from the same source; (4) studies containing fewer than 20 patients; (5) studies on pleural effusion from parapneumonic, heart failure-related, malignant, or tuberculous diseases; (6) PE diagnosis strictly based on ventilation/perfusion (WQ) scanning or autopsy; (7) published only in abstract form; and (8) not published in English. Finally, if only severe PE was included or the method for evaluating pleural effusion was invasive, the article was excluded.

Two independent investigators conducted a systematic search based on the inclusion criteria, and screened the titles and abstracts of the identified studies to exclude duplicates or irrelevant records. For articles requiring further evaluation, a full text review was conducted and references to retrieved articles and related reviews were manually checked to identify other eligible studies, with disagreements resolved by a third researcher.

### 2.3. Data Extraction and Quality Assessment

A standardized Excel (Microsoft Corporation) file was used by two authors (PL and JA) who independently extracted data from selected studies in duplicate concerning the following: author, publication year, country, study design, inclusion criteria, exclusion criteria, number of subjects (PE with pleural effusion and PE), participant demographic data, mean age, sex, methods of assessment of pleural effusion, follow-up after discharge, and short-term mortality (30-day and in-hospital mortality). Where data were unavailable, the original author of the study was contacted by e-mail. The quality of each individual study was independently evaluated by two authors using the Newcastle-Ottawa Scale (NOS) [16], which included selection (4 items), comparability (1 item), and outcome (3 items) with three levels (low quality = 0–3, moderate quality = 4–6, and high quality = 7–9, respectively). For any inconsistencies between the authors in data extraction and quality assessment among the authors, a consensus was obtained through discussion with a third reviewer.

### 2.4. Statistical Analysis

The incidence of pleural effusion in PE was expressed as proportions with 95% confidence intervals (CIs) and displayed as a forest plot using the random-effects model that accounted for the potential variation among the included studies [17]. Considering that the location and size of pleural effusion in PE is still controversial, the incidence of unilateral pleural effusion and small pleural effusion in PE was also analyzed [18,19]. In addition, we performed a meta-analysis to assess the effect of pleural effusion on PE prognosis for a subset of comparable studies. The results were reported as risk ratios (RRs) with 95% confidence intervals (CIs). Heterogeneity among the studies was determined using the Q-test and the I-squared (I^2^) test. When significant heterogeneity (I^2^ > 50%) was detected, the random-effects model was employed for the meta-analysis. Otherwise, the fixed-effects model was employed. Subgroup analysis (geographical location, assessment of pleural effusion, study design, mortality) was performed to investigate heterogeneity, and sensitivity analysis (leave-one-out analysis) was used to examine the stability of our results. Funnel plots and Begg’s and Egger’s tests were used to analyze publication bias, and *p* < 0.05 was considered to be statistically significant. Statistical analysis was conducted in Stata (version 12.0).

## 3. Results

### 3.1. Study Selection and Characteristics

**Figure 1:**
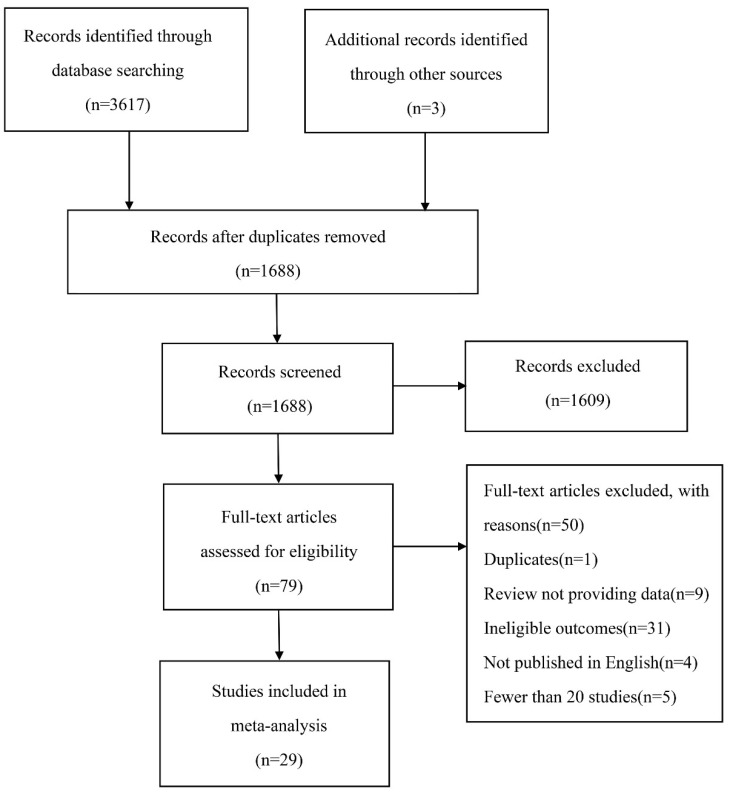
The flow diagram of study selection.

The literature search yielded 3620 articles. After removing duplicates, 1688 articles were screened for eligibility based on their titles and abstracts; of these, 1609 articles did not meet our inclusion criteria and were excluded. The remaining 79 articles were screened for full-text, of which 50 were removed for various reasons, including duplicates (1 article), review not providing data (9 articles), ineligible outcomes (31 articles), not published in English (4 articles) and fewer than 20 patients (5 articles). Finally, 29 articles were included for data extraction (Figure 1). The number of patients with PE was 13,430, among which the incidence of pleural effusion with PE was more than 30.4%. The sample sizes of these studies ranged from 22 to 3391 and were published between 1978 and 2022. Among the included studies, 29 examined the incidence of pleural effusion in PE, of which 6 were prospectively designed, while the remaining 23 were retrospective [18,19,20,21,22,23,24,25,26,27,28,29,30,31,32,33,34,35,36,37,38,39,40,41,42,43,44,45,46]. Data on short-term mortality (30-day or in-hospital mortality) in PE patients with or without pleural effusion were available in 7 cohort studies [40,41,42,43,44,45,46]. Moreover, 9 studies were carried out in Europe, 7 in Asia, 6 in North America, 5 in Europe and Asia, and 2 in multiple states. The characteristics of the included studies are listed in Table 1.

### 3.2. Risk of Bias

Table S2 in the Supplementary Materials displays the results of the quality assessment of the 29 studies using the Newcastle-Ottawa Scale. Among the 29 studies included in the analysis, the Newcastle–Ottawa score range was 6 to 8 (maximum 8), with higher scores indicating a lower risk of bias. All included studies were rated as high quality with a total score of ≥ 6 (Table S2), indicating a low risk of bias.

### 3.3. Pleural Effusion in PE

**Figure 2:**
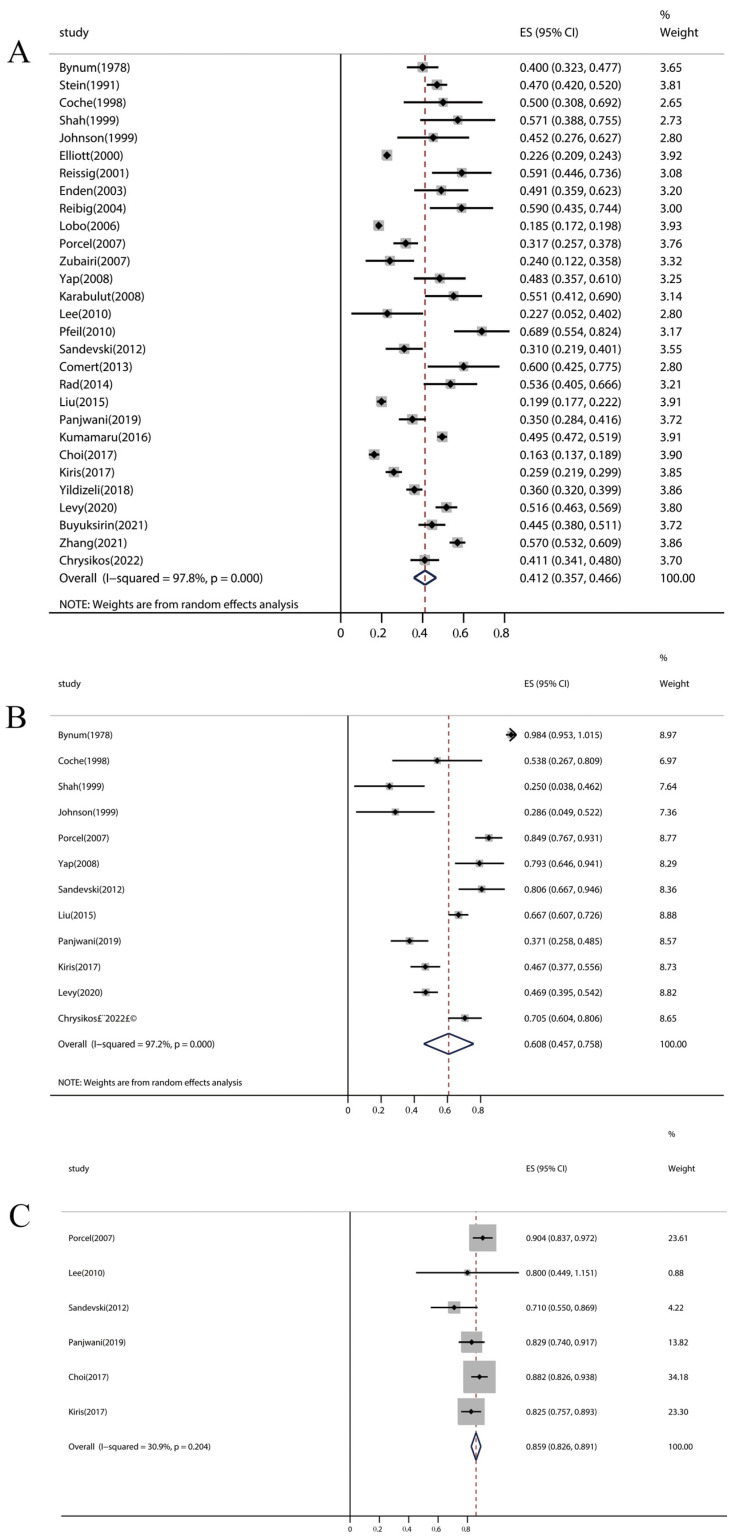
Forest plot of pleural effusion incidence in PE patients. (**A**) Incidence of pleural effusion. (**B**) Incidence of unilateral pleural effusion. (**C**) Incidence of small pleural effusion. PE, pulmonary embolism.

The overall pooled incidence of pleural effusion among PE patients was 41.2% (95% CI: 35.7–46.6%, *p* < 0.001), and there was considerable heterogeneity in the effect estimate among studies (I^2^ = 97.8%, *p* < 0.001) (Figure 2A). Further exclusion of any signal study did not significantly alter the overall pooled estimate, which ranged from 40.2% to 42.2%. Only twelve [18,19,20,22,23,24,30,33,36,39,41,44] and six [19,30,34,36,41,42] articles (out of 29 articles) examined the location and size of pleural effusion in PE, respectively. The pooled incidence of unilateral pleural effusion and small pleural effusion was 60.8% (95% CI: 45.7–75.8%, *p* < 0.001) (Figure 2B) and 85.9% (95% CI: 82.6–89.1%, *p* < 0.001) (Figure 2C) among PE patients, respectively. Subgroup analyses indicated that the potential main causes of heterogeneity were geographical location (*p* < 0.001), methods of pleural effusion assessment (*p* < 0.001), and study design (*p* < 0.001) (Table 2). Moreover, the subgroup analysis revealed some findings: North America had the highest incidence of pleural effusion in PE patients among the four locations (North America, Europe, Asia, and Europe and Asia), and the pooled incidence of pleural effusion detected by transthoracic sonography (TS) in PE was higher than that detected by computed tomography (CT) or chest X-ray (CXR).

### 3.4. Association of Pleural Effusion with PE Patient Outcome

**Figure 3:**
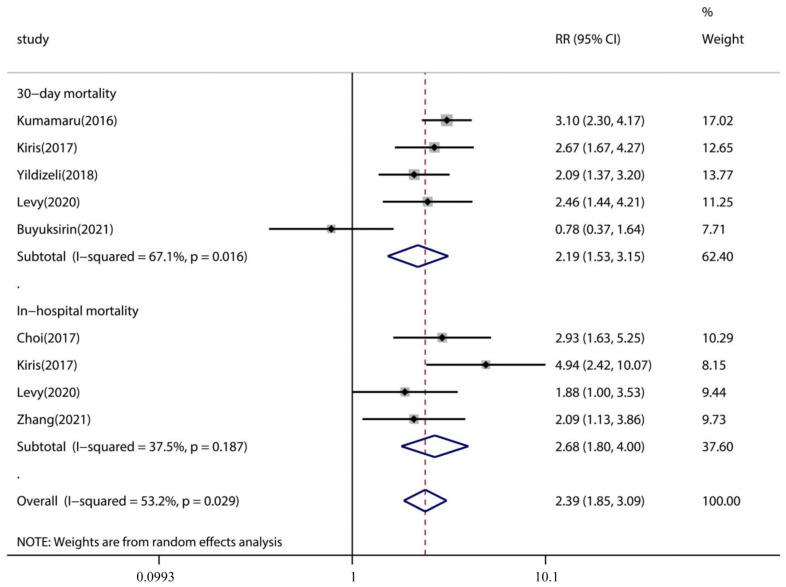
Descriptive forest plot of the effect of pleural effusion on PE 30-day mortality and in-hospital mortality outcomes. CI, confidence interval; PE, pulmonary embolism.

Only seven studies explored the association between pleural effusion and mortality risk in PE patients. Subgroup analyses demonstrated that the pooled incidence of 30-day mortality was significantly higher in PE with pleural effusion (pooled incidence: 18.9%, 95% CI: 14.9–22.9%) than in PE without pleural effusion (pooled incidence: 8.6%, 95% CI: 6.4–10.8%) (*p* < 0.001) (Table 3). The same was true for in-hospital mortality of PE with pleural effusion (pooled incidence: 12.0%, 95% CI: 9.7–14.2%) compared with PE without pleural effusion (pooled incidence: 4.3%, 95% CI: 3.2–5.3%) (*p* < 0.001) (Table 3). Pooled analysis using a random-effects model (I^2^ = 53.2%) showed that pleural effusion was linked with an increased risk of 30-day mortality (RR 2.19, 95% CI: 1.53–3.15, *p* < 0.001, I^2^ = 67.1%) and in-hospital mortality (RR 2.39, 95% CI: 1.85–3.09, *p* < 0.001, I2 = 37.1%) in patients with PE (Figure 3).

### 3.5. Assessment of Sensitivity Analysis and Bias

**Figure 4:**
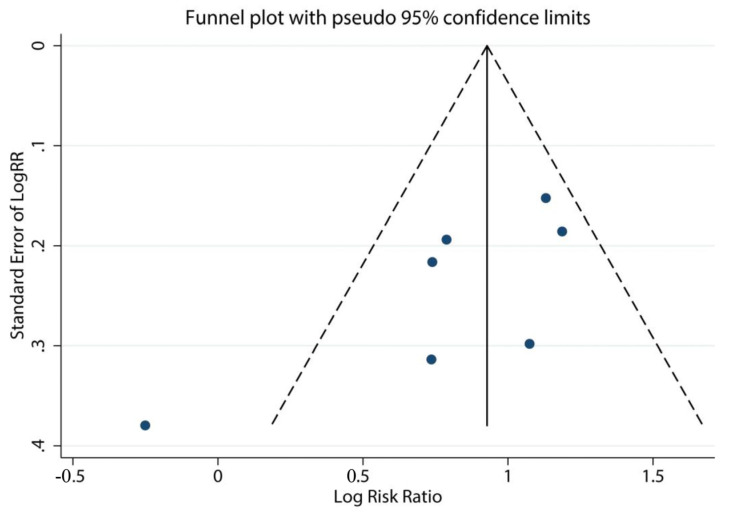
Sensitivity analysis of pleural effusion and the risk of death in PE. PE, pulmonary embolism.

**Figure 5:**
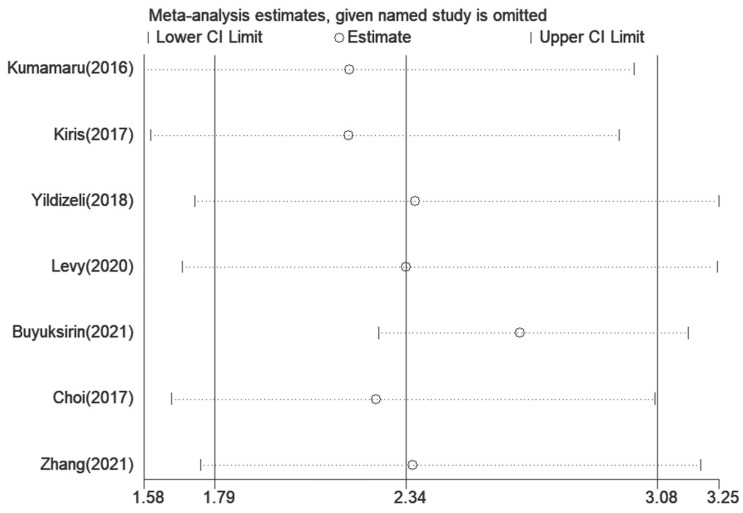
The funnel plot of pleural effusion and the risk of death in PE. PE, pulmonary embolism.

A sensitivity analysis was conducted only among the seven articles [40,41,42,43,44,45,46] concerning the association between pleural effusion and the mortality risk in PE. After removing any study individually, the meta-analysis results did not substantially differ from those obtained with all studies (Figure 4). Potential publishing bias was noted by using a funnel plot (Figure 5), but neither Begg’s test (*p* = 0.072) nor Egger’s test (*p* = 0.071) indicated a significant risk of publication bias, which may be due to the limited number of articles.

## 4. Discussion

The incidence of pleural effusion varies among PE patients, and whether pleural effusion could increase the mortality rate remains inconclusive. This systematic review and meta-analysis identified 29 observational studies with 13,430 subjects that assessed the incidence of pleural effusion in PE and 7 studies among 29 explored the association between pleural effusion and PE mortality. The pooled incidence of pleural effusion in PE was up to 41.2% (95% CI: 35.7–46.6%), which was affected by geographical location, methods of pleural effusion assessment, and study design. Moreover, PE patients were susceptible to developing unilateral and small pleural effusions, which is consistent with previous findings [10,30]. In addition, pleural effusion also increased the risk of death from PE, including 30-day mortality and in-hospital mortality, compared with pure PE.

Early detection and treatment of PE can improve the prognosis and survival rate [47]. Unfortunately, due to the absence of distinct clinical signs and symptoms of PE, especially when combined with comorbidities such as pleural effusion, it can be overlooked or misdiagnosed [12,48]. Studies have shown that pleural effusion has a high incidence in PE and that its incidence has a wide distribution of values [19,44]. A review by Agarwal et al. [49] illustrated that the pooled incidence of pleural effusion in PE patients was 19~61%, which was roughly consistent with our findings. The present study analyzed existing studies on pleural effusion in PE with an incidence ranging from 16.3 to 68.9% in each study, and the pooled incidence of pleural effusion in PE was as high as 41.2%, which was generally consistent with previous studies [10]. The incidence is highest if TS is used to detect pleural effusion (59.3% with TS, 43.0% with CT, and 30.5% with CXR), which confirms the results of a study by Agarwal et al. [49]. Meanwhile, as the results of the present study suggest, pleural effusion in PE is typically small and unilateral, so the majority of patients cannot undergo thoracentesis, potentially increasing diagnostic difficulty, and its true prevalence in PE may be higher [11,19], necessitating more attention in clinical activities regarding the differential diagnosis of an undiagnosed pleural effusion in PE. The present study indicated that variability in individual studies can be partially explained by differences in geographic location, assessment methods, and study design, which demonstrated that, as potential sources of bias, these may bring apparent errors, confounding the true incidence of pleural effusion in patients with PE. It is also unfortunate that although we undertook a comprehensive literature search, we were unable to find relevant data in the literature on pleural effusion according to PE severity that might provide more insights into this question.

Given the prevalence of pleural effusion, it may be surprising that relatively few studies have investigated its impact on PE patients. With recently published papers, our study provides important information to the previous meta-analysis by Zuin et al. [50]. This meta-analysis included 7 articles, which examined the impact of pleural effusion on short-term mortality (30-day and in-hospital mortality) in PE patients, comprising 4707 PE patients (n = 1930 with pleural effusion); a proportion, but not all, of the included articles overlapped due to differences in eligibility criteria. Zuin et al. suggested that pleural effusion was significantly associated with a higher risk of death in the short-term period, which is consistent with our results. Our sensitivity analysis confirms the association between pleural effusion and short-term mortality in PE patients. Moreover, the studies on the effect of pleural effusion on PE identified in this review [40,41,42,43,44,45,46] are recent and demonstrate growing concern about the effect of pleural effusion on the prognosis of patients with PE. Despite the limited number of studies, the report shows that the association between pleural effusion and mortality in patients with PE is compelling given the large sample of patients with PE (n = 4707), with a low risk of bias across studies. In addition, our exploratory meta-analysis revealed an increased risk of 30-day and in-hospital mortality when pleural effusion occurred in PE. Overall, this review suggests that the incidence of pleural effusion is very high, which increases short-term mortality (30-day and in-hospital mortality) in PE patients. According to a Chinese study, pleural effusion is an independent risk factor for poor prognosis in patients with PE, and the all-cause mortality of PE patients with pleural effusion was significantly higher than that of PE patients without pleural effusion at both the 3-month and 1-year follow-up points [12]. Pulmonary infarction syndrome and circulatory collapse are among the most prevalent clinical presentations in PE patients with pleural effusion [19,29], and the occurrence of pleural effusion is thought to be associated with a longer hospital stay, severity and prognosis of PE [45,51]. However, the mechanism by which pleural effusion affects the condition in PE remains unclear. Significant underlying comorbidities [12,43], such as right ventricular (RV) failure, may account for the increased mortality risk associated with pleural effusion in PE patients. The pathogenetic underlying mechanism of pleural effusion secondary to PE is the increased permeability of pulmonary capillaries [10,52]. The increased systemic venous pressure on the parietal pleural surface as a result of pulmonary hypertension and increased right ventricular (RV) pressure may account for the increased permeability [11]. Moreover, previous studies have shown that patients with pulmonary hypertension and right heart failure frequently exhibit pleural effusions. Patients should be evaluated for pleural effusion when pulmonary hypertension is present and, if present, should be examined for right heart failure [53]. RV enlargement is predictive of poor prognosis, including PE-related in-hospital shock, the need for cardiopulmonary resuscitation, and PE-related mortality, which is considered the leading cause of death from severe PE [4,54]. Therefore, we speculate that pleural effusion is strongly associated with the progression of RV failure, which further contributes to poorer outcomes in PE. Consequently, although pleural effusion does not alter the standard treatment for PE [11], it should be regarded as a major warning sign of an increased risk of death in PE patients, which must be confirmed by additional research.

While compelling, the data should be interpreted cautiously owing to the heterogeneity in the design, setting, and inclusion criteria of the included studies. However, several limitations of the present study must be considered. First, this study relies solely on observational data, which are very susceptible to selection bias and confounding by indication. Second, heterogeneity analysis indicated that our results were highly heterogeneous, although we performed subgroup analysis to partially explain and reduce this. Third, there was inconsistent data collection, and the characteristics of the subjects were unclear, all of which could have affected the results, leaving residual bias, such as disease severity and comorbidity-related bias. Fourth, the number of studies on the risk of death in pleural effusion with PE was limited; thus, the results need to be carefully understood. Fifth, the study was not registered. Finally, it is worth noting that the large number of patients included in the meta-analysis allowed reasonable effect estimates, but data on potentially relevant confounders were available only in some studies (malignancy, cardiac disease, and parapneumonia), which led to potential bias.

The strength of this systematic review is that it addresses the incidence of pleural effusion, a very common clinical comorbidity in PE, which may have a significant impact on PE patient outcome. Our study shows that pleural effusion has a high incidence in PE patients and is associated with the prognosis of PE patients, with most studies finding an opposite impact on the outcomes of PE patients.

## 5. Conclusions

In conclusion, this systematic review and meta-analysis determined that the pooled incidence of pleural effusion was 41.2% (95% CI: 35.6–46.8%). Additionally, pleural effusion generally increased short-term mortality (30-day and in-hospital mortality) in PE patients. We recommend that physicians be aware of the risk of death from PE, especially when patients have pleural effusion. To better inform policy and practice, more research is required to determine which clinical and demographic factors can regulate this increased risk.

## Figures and Tables

**Table 1:** Characteristics of included studies.

Study/Year	Study Design	Country	No. of PE Patients/Pleural Effusion in PE	Short-Term Mortality	Location of Pleural Effusion (Unilateral/Bilateral)	Size of Pleural Effusion (Small/Moderate /Large)	Assessment of Pleural Effusion
Bynum (1978)	Prospective	USA	155/62	NA	61/1	NA/NA/NA	CXR
Stein (1991)	Prospective	USA	383/180	NA	NA/NA	NA/NA/NA	CXR
Coche (1998)	Retrospective	British	26/13	NA	7/6	NA/NA/NA	CT
Shah (1999)	Retrospective	USA	28/16	NA	4/12	NA/NA/NA	CT
Johnson (1999)	Retrospective	USA	31/14	NA	4/10	NA/NA/NA	CT
Elliott (2000)	Prospective	Europe and North America	2319/523	NA	NA/NA	NA/NA/NA	CXR
Reissig (2001)	Prospective	Germany	44/26	NA	NA/NA	NA/NA/NA	TS
Enden (2003)	Retrospective	Norway	55/27	NA	NA/NA	NA/NA/NA	CT
Reibig (2004)	Retrospective	Germany	39/23	NA	NA/NA	NA/NA/NA	TS
Lobo (2006)	Prospective	Spain, France, Italy, Israel, Argentina	3391/628	NA	NA/NA	NA/NA/NA	CXR
Porcel (2007)	Retrospective	Spain	230/73	NA	62/11	66/3/4	CXR
Zubairi (2007)	Retrospective	Pakistan	50/12	NA	NA/NA	NA/NA/NA	CXR
Yap (2008)	Retrospective	UK	60/29	NA	23/6	NA/NA/NA	CT
Karabulut (2008)	Retrospective	Turkey	49/27	NA	NA/NA	NA/NA/NA	CT
Lee (2010)	Retrospective	USA	22/5	NA	NA/NA	4/1/0	CT
Pfeil (2010)	Retrospective	Germany	45/31	NA	NA/NA	NA/NA/NA	CT
Sandevski (2012)	Retrospective	Macedonia	100/31	NA	25/6	22/8/1	CXR
Comert (2013)	Prospective	Turkey	30/18	NA	NA/NA	NA/NA/NA	TS
Rad (2014)	Retrospective	Iran	56/30	NA	NA/NA	NA/NA/NA	CT
Liu (2015)	Retrospective	China	1220/243	NA	162/81	NA/NA/NA	CT
Panjwani (2019)	Retrospective	Bahrain	200/70	NA	26/44	58/9/3	CT
Chrysikos (2022)	Retrospective	Greece	190/78	NA	55/23	NA/NA/NA	CT
Kumamaru (2016)	Retrospective	United States	1698/841	30-day mortality (210)	NA/NA	NA/NA/NA	CT
Choi (2017)	Retrospective	Korea	778/127	In-hospital mortality (44)	NA/NA	112/13/2	CT
Kiris (2017)	Retrospective	Turkey	463/120	30-day mortality (58), In-hospital mortality (30)	56/64	99/20/1	CT
Yildizeli (2018)	Retrospective	Turkey	570/205	30-day mortality (74)	NA/NA	NA/NA/NA	CT
Levy (2020)	Retrospective	Israel	343/177	30-day mortality (58), In-hospital mortality (39)	83/94	NA/NA/NA	CT
Buyuksirin (2021)	Retrospective	Turkey	220/98	30-day mortality (26)	NA/NA	NA/NA/NA	CT
Zhang (2021)	Retrospective	China	635/362	In-hospital mortality (49)	NA/NA	NA/NA/NA	CT

Abbreviations: TS, transthoracic sonography; CXR, chest X-ray; CT, computed tomography.

**Table 2:** Subgroup analyses regarding risk of pleural effusion.

Subgroups	No. Studies	Pooled Estimate (%)	95% CI	*p* Value	I^2^
Geographic location					
North America	6	45.1	39.5–50.6	*p* < 0.001	66.5%
Europe	9	47.5	39.0–56.1	*p* < 0.001	82.1%
Asia	7	36.6	22.9–50.3	*p* < 0.001	98.6%
Europe and Asia	5	41.9	32.2–51.7	*p* < 0.001	90.9%
Methods of pleural effusion assessment					
TS	3	59.3	50.2–68.4	*p* < 0.001	0.0%
CXR	7	30.5	24.0–37.1	*p* < 0.001	96.2%
CT	19	43.0	35.3–50.7	*p* < 0.001	97.7%
Study design					
Prospective	6	38.0	29.7–46.4	*p* < 0.001	97.3%
Retrospective	23	41.7	34.9–48.6	*p* < 0.001	97.2%

Abbreviations: TS, transthoracic sonography; CXR, chest X-ray; CT, computed tomography.

**Table 3:** Subgroup analysis of mortality.

Subgroups	No. Studies	Pooled Estimate (%)	95% CI (%)	*p* Value	I^2^
30-day mortality					
PE with pleural effusion	5	18.9	14.9–22.9	*p* < 0.001	65.9%
PE	5	8.6	6.4–10.8	*p* < 0.001	57.5%
In-hospital mortality					
PE with pleural effusion	4	12.0	9.7–14.2	*p* < 0.001	26.7%
PE	4	4.3	3.2–5.3	*p* < 0.001	30.4%

Abbreviations: PE, pulmonary embolism.

## Data Availability

Not applicable.

## Supplementary Materials

The following supporting information can be downloaded at: https://www.mdpi.com/article/10.3390/jcm12062315/s1, Table S1: MOOSE checklist for Meta-analyses of Observational Studies; Table S2: Quality assessment of the included studies.

## References

[1] Hepburn-Brown M., Darvall J., Hammerschlag G. (2019). Acute pulmonary embolism: A concise review of diagnosis and management. Intern. Med. J..

[2] Alikhan R., Peters F., Wilmott R., Cohen A.T. (2004). Fatal pulmonary embolism in hospitalised patients: A necropsy review. J. Clin. Pathol..

[3] Bikdeli B., Wang Y., Jimenez D., Parikh S.A., Monreal M., Goldhaber S.Z., Krumholz H.M. (2019). Pulmonary Embolism Hospitalization, Readmission, and Mortality Rates in US Older Adults, 1999-2015. JAMA.

[4] Konstantinides S.V., Meyer G., Becattini C., Bueno H., Geersing G.J., Harjola V.P., Huisman M.V., Humbert M., Jennings C.S., Jiménez D. (2020). 2019 ESC Guidelines for the diagnosis and management of acute pulmonary embolism developed in collaboration with the European Respiratory Society (ERS). Eur. Heart J..

[5] Furfaro D., Stephens R.S., Streiff M.B., Brower R. (2018). Catheter-directed Thrombolysis for Intermediate-Risk Pulmonary Embolism. Ann. Am. Thorac. Soc..

[6] Lee T., Itagaki S., Chiang Y.P., Egorova N.N., Adams D.H., Chikwe J. (2018). Survival and recurrence after acute pulmonary embolism treated with pulmonary embolectomy or thrombolysis in New York State, 1999 to 2013. J. Thorac. Cardiovasc. Surg..

[7] Porcel J.M., Light R.W. (2008). Pleural effusions due to pulmonary embolism. Curr. Opin. Pulm. Med..

[8] Light R.W. (2002). Clinical practice. Pleural effusion. N. Engl. J. Med..

[9] Jany B., Welte T. (2019). Pleural Effusion in Adults—Etiology, Diagnosis, and Treatment. Dtsch. Arztebl. Int..

[10] Light R.W. (2010). Pleural effusion in pulmonary embolism. Semin. Respir. Crit. Care Med..

[11] Findik S. (2012). Pleural effusion in pulmonary embolism. Curr. Opin. Pulm. Med..

[12] Zhou X., Zhang Z., Zhai Z., Zhang Y., Miao R., Yang Y., Xie W., Wan J., Wang C. (2016). Pleural effusions as a predictive parameter for poor prognosis for patients with acute pulmonary thromboembolism. J. Thromb. Thrombolysis.

[13] Calder K.K., Herbert M., Henderson S.O. (2005). The mortality of untreated pulmonary embolism in emergency department patients. Ann. Emerg. Med..

[14] Stroup D.F., Berlin J.A., Morton S.C., Olkin I., Williamson G.D., Rennie D., Moher D., Becker B.J., Sipe T.A., Thacker S.B. (2000). Meta-analysis of observational studies in epidemiology: A proposal for reporting. Meta-analysis Of Observational Studies in Epidemiology (MOOSE) group. JAMA.

[15] Page M.J., McKenzie J.E., Bossuyt P.M., Boutron I., Hoffmann T.C., Mulrow C.D., Shamseer L., Tetzlaff J.M., Akl E.A., Brennan S.E. (2021). The PRISMA 2020 statement: An updated guideline for reporting systematic reviews. BMJ.

[16] Wells G., Shea B., O’Connell D., Peterson J., Welch V., Losos M., Tugwell P. (2021). The Newcastle-Ottawa Scale (NOS) for Assessing the Quality of Nonrandomised Studies in Meta-Analyses. https://www.ohri.ca/programs/clinical_epidemiology/oxford.asp.

[17] Ades A.E., Lu G., Higgins J.P. (2005). The interpretation of random-effects meta-analysis in decision models. Med. Decis. Mak. Int. J. Soc. Med. Decis. Mak..

[18] Chrysikos S., Papaioannou O., Karampitsakos T., Tavernaraki K., Thanou I., Filippousis P., Anyfanti M., Hillas G., Tzouvelekis A., Thanos L. (2022). Diagnostic Accuracy of Multiple D-Dimer Cutoff Thresholds and Other Clinically Applicable Biomarkers for the Detection and Radiographic Evaluation of Pulmonary Embolism. Adv. Respir. Med..

[19] Panjwani A., Zaid T., Alawi S., Al Shehabi D., Abdulkarim E.S. (2019). Pleural effusion in acute pulmonary embolism in Bahrain: Radiological and pleural fluid characteristics. Lung India.

[20] Bynum L.J., Wilson J.E. (1978). Radiographic features of pleural effusions in pulmonary embolism. Am. Rev. Respir. Dis..

[21] Stein P.D., Saltzman H.A., Weg J.G. (1991). Clinical characteristics of patients with acute pulmonary embolism. Am. J. Cardiol..

[22] Coche E.E., Müller N.L., Kim K.I., Wiggs B.R., Mayo J.R. (1998). Acute pulmonary embolism: Ancillary findings at spiral CT. Radiology.

[23] Johnson P.T., Wechsler R.J., Salazar A.M., Fisher A.M., Nazarian L.N., Steiner R.M. (1999). Spiral CT of acute pulmonary thromboembolism: Evaluation of pleuroparenchymal abnormalities. J. Comput. Assist. Tomogr..

[24] Shah A.A., Davis S.D., Gamsu G., Intriere L. (1999). Parenchymal and pleural findings in patients with and patients without acute pulmonary embolism detected at spiral CT. Radiology.

[25] Elliott C.G., Goldhaber S.Z., Visani L., DeRosa M. (2000). Chest radiographs in acute pulmonary embolism. Results from the International Cooperative Pulmonary Embolism Registry. Chest.

[26] Reissig A., Heyne J.P., Kroegel C. (2001). Sonography of lung and pleura in pulmonary embolism: Sonomorphologic characterization and comparison with spiral CT scanning. Chest.

[27] Enden T., Kløw N.E. (2003). CT pulmonary angiography and suspected acute pulmonary embolism. Acta Radiol..

[28] Reibig A., Heyne J.-P., Kroegel C. (2004). Ancillary lung parenchymal findings at spiral CT scanning in pulmonary embolism. Relationship to chest sonography. Eur. J. Radiol..

[29] Lobo J.L., Zorrilla V., Aizpuru F., Uresandi F., Garcia-Bragado F., Conget F., Monreal M. (2006). Clinical syndromes and clinical outcome in patients with pulmonary embolism: Findings from the RIETE registry. Chest.

[30] Porcel J.M., Madronero A.B., Pardina M., Vives M., Esquerda A., Light R.W. (2007). Analysis of pleural effusions in acute pulmonary embolism: Radiological and pleural fluid data from 230 patients. Respirology.

[31] Zubairi A.B., Husain S.J., Irfan M., Fatima K., Zubairi M.A., Islam M. (2007). Chest radiographs in acute pulmonary embolism. JAMC: J. Ayub Med. Coll. Abbottabad..

[32] Karabulut N., Kiroğlu Y. (2008). Relationship of parenchymal and pleural abnormalities with acute pulmonary embolism: CT findings in patients with and without embolism. Diagn. Interv. Radiol..

[33] Yap E., Anderson G., Donald J., Wong C.A., Lee Y.C., Sivakumaran P. (2008). Pleural effusion in patients with pulmonary embolism. Respirology.

[34] Lee E.Y., Zurakowski D., Diperna S., d’Almeida Bastos M., Strauss K.J., Boiselle P.M. (2010). Parenchymal and pleural abnormalities in children with and without pulmonary embolism at MDCT pulmonary angiography. Pediatr. Radiol..

[35] Pfeil A., Schmidt P., Hermann R., Bottcher J., Wolf G., Hansch A. (2010). Parenchymal and pleural findings in pulmonary embolism visualized by multi-channel detector computed tomography. Acta Radiol..

[36] Sandevski A., Jovkovska Kaeva B., Gligorovski L., Simonovska L., Sandevska E. (2012). Frequency and characteristics of pleural effusions in pulmonary embolism. Contrib. Sec. Biol. Med. Sci. MASA.

[37] Comert S.S., Caglayan B., Akturk U., Fidan A., Kiral N., Parmaksiz E., Salepci B., Kurtulus B.A. (2013). The role of thoracic ultrasonography in the diagnosis of pulmonary embolism. Ann. Thorac. Med..

[38] Rad M.P., Tehrani D.F., Reihani H., Sabzevari S.H.F., Rajabi M. (2014). Incidental Findings in Patients Evaluated for Pulmonary Embolism Using Computed Tomography Angiography. J. Cardio-Thorac. Med..

[39] Liu M., Cui A., Zhai Z.G., Guo X.J., Li M., Teng L.L., Xu L.L., Wang X.J., Wang Z., Shi H.Z. (2015). Incidence of pleural effusion in patients with pulmonary embolism. Chin. Med. J..

[40] Kumamaru K.K., Saboo S.S., Aghayev A., Cai P., Quesada C.G., George E., Hussain Z., Cai T., Rybicki F.J. (2016). CT pulmonary angiography-based scoring system to predict the prognosis of acute pulmonary embolism. J. Cardiovasc. Comput. Tomogr..

[41] Kiris T., Yazici S., Koc A., Koprulu C., Ilke Akyildiz Z., Karaca M., Nazli C., Dogan A. (2017). Prognostic impact of pleural effusion in acute pulmonary embolism. Acta Radiol..

[42] Choi S.H., Cha S.I., Shin K.M., Lim J.K., Yoo S.S., Lee S.Y., Lee J., Kim C.H., Park J.Y., Lee D.H. (2017). Clinical Relevance of Pleural Effusion in Patients with Pulmonary Embolism. Respiration.

[43] Yildizeli S.O., Kasapoglu U.S., Arikan H., Cimsit C., Cimsit N.C., Suzer Aslan M., Kocakaya D., Eryuksel E., Ceyhan B., Karakurt S. (2018). Pleural effusion as an indicator of short term mortality in acute pulmonary embolism. Tuberk Toraks.

[44] Levy O., Fux D., Bartsikhovsky T., Vosko S., Tishler M., Copel L. (2020). Clinical relevance of bilateral pleural effusion in patients with acute pulmonary embolism. Intern. Med. J..

[45] Zhang J., Zhou H., Aili A., Wang M., Shen Y., Yi Q. (2021). Prevalence and clinical significance of pleural effusion in patients with acute pulmonary embolism: A retrospective study. J. Thorac. Dis..

[46] Buyuksirin M., Anar C., Polat G., Karadeniz G. (2021). Can the Level of CRP in Acute Pulmonary Embolism Determine Early Mortality?. Turk. Thorac. J..

[47] Roy P.M., Meyer G., Vielle B., Le Gall C., Verschuren F., Carpentier F., Leveau P., Furber A. (2006). Appropriateness of diagnostic management and outcomes of suspected pulmonary embolism. Ann. Intern. Med..

[48] Van Maanen R., Trinks-Roerdink E.M., Rutten F.H., Geersing G.J. (2022). A systematic review and meta-analysis of diagnostic delay in pulmonary embolism. Eur. J. Gen. Pract..

[49] Agarwal R., Singh N., Gupta D. (2009). Pleural Effusions Associated with Pulmonary Thromboembolism: A Systematic Review. Indian J. Chest Dis. Allied Sci..

[50] Zuin M., Rigatelli G., Turchetta S., Pasquetto G., Roncon L., Bilato C. (2022). Mortality Risk in Patients with Pulmonary Embolism with Pleural Effusion. Am. J. Cardiol..

[51] Heyer C.M., Lemburg S.P., Knoop H., Holland-Letz T., Nicolas V., Roggenland D. (2011). Multidetector-CT angiography in pulmonary embolism-can image parameters predict clinical outcome?. Eur. Radiol..

[52] Wiener-Kronish J.P., Broaddus V.C., Albertine K.H., Gropper M.A., Matthay M.A., Staub N.C. (1988). Relationship of pleural effusions to increased permeability pulmonary edema in anesthetized sheep. J. Clin. Investig..

[53] Brixey A.G., Light R.W. (2011). Pleural effusions occurring with right heart failure. Curr. Opin. Pulm. Med..

[54] Alerhand S., Sundaram T., Gottlieb M. (2021). What are the echocardiographic findings of acute right ventricular strain that suggest pulmonary embolism?. Anaesth. Crit. Care Pain Med..

