# Supplementary material for: Incidence and Prognostic Role of Pleural Effusion in Patients with Pulmonary Embolism: A Systematic Review and Meta-Analysis
Source: J Clin Med. 2023 Mar 16;12(6):2315. doi: 10.3390/jcm12062315 (PMC10058137; doi:10.3390/jcm12062315)
Supplement: Supplementary file 1 [file jcm-12-02315-s001.zip › Supplementary tables.pdf]

Table S1. MOOSE checklist for Meta-analyses of Observational Studies

Item No	Recommendation	Reported section
Reporting of background should include		
1	Problem definition	Introduction
2	Hypothesis statement	Introduction
3	Description of study outcome(s)	Introduction
4	Type of exposure or intervention used	Introduction
5	Type of study designs used	Introduction
6	Study population	Introduction
Reporting of search strategy should include		
7	Qualifications of searchers (eg, librarians and investigators)	Methods
8	Search strategy, including time period included in the synthesis and keywords	Methods
9	Effort to include all available studies, including contact with authors	Methods
10	Databases and registries searched	
11	Search software used, name and version, including special features used (eg, explosion)	Methods
12	Use of hand searching (eg, reference lists of obtained articles)	Methods
13	List of citations located and those excluded, including justification	Fig1
14	Method of addressing articles published in languages other than English	N/A
15	Method of handling abstracts and unpublished studies	Methods
16	Description of any contact with authors	N/A
Reporting of methods should include		
17	Description of relevance or appropriateness of studies assembled for assessing the hypothesis to be tested	Methods
18	Rationale for the selection and coding of data (eg, sound clinical principles or convenience)	Methods
19	Documentation of how data were classified and coded (eg, multiple raters, blinding, and interrater reliability)	Methods
20	Assessment of confounding (eg, comparability of cases and controls in studies where appropriate)	Methods
21	Assessment of study quality, including blinding of quality assessors; stratification or regression on possible predictors of study results	Methods
22	Assessment of heterogeneity	Methods

23	Description of statistical methods (eg, complete description of fixed or random effects models, justification of whether the chosen models account for predictors of study results, dose-response models, or cumulative meta-analysis) in sufficient detail to be replicated	Methods
24	Provision of appropriate tables and graphics	Methods and Results
Reporting of results should include		
25	Graphic summarizing individual study estimates and overall estimate	Fig2,3
26	Table giving descriptive information for each study included	TableS2
27	Results of sensitivity testing (eg, subgroup analysis)	Table1, Fig5
28	Indication of statistical uncertainty of findings	Results
Reporting of discussion should include		
29	Quantitative assessment of bias (eg, publication bias)	Discussion, Fig4
30	Justification for exclusion (eg, exclusion of non–English-language citations)	Discussion
31	Assessment of quality of included studies	Results, TableS2
Reporting of conclusions should include		
32	Consideration of alternative explanations for observed results	Discussion
33	Generalization of the conclusions (ie, appropriate for the data presented and within the domain of the literature review)	Discussion, Conclusion
34	Guidelines for future research	Discussion, Conclusion
35	Disclosure of funding source	Acknowledgements

Stroup DF, Berlin JA, Morton SC, et al. Meta-analysis of observational studies in epidemiology: a proposal for reporting. Meta-analysis Of Observational Studies in Epidemiology (MOOSE) group. *JAMA*. 2000;283(15):2008-2012.

Table S2. Quality assessment of the included studies

Study/Year	Selection				Comparability	Outcome			Total score
	Representativeness of the exposed cohort	Selection of the non-exposed cohort	Ascertainment of exposure	Demonstration that outcome of interest was not present at start of study	Comparability of cohorts on the basis of the design or analysis	Assessment of outcome	Was follow-up long enough for outcomes to occur	Adequacy of follow-up of cohorts	
Bynum(1978)	1	1	1	0	1	1	1	1	7
Stein(1991)	1	1	1	0	0	1	1	1	6
Coche(1998)	1	1	1	0	1	1	1	1	7
Shah(1999)	1	1	1	0	1	1	1	1	7
Johnson(1999)	1	1	1	0	0	1	1	1	6
Elliott(2000)	1	1	1	0	1	1	1	1	7
Reissig(2001)	1	1	1	0	0	1	1	1	6
Enden(2003)	1	1	1	0	1	1	1	1	7
Reibig(2004)	1	1	1	0	0	1	1	1	6
Lobo(2006)	1	1	1	0	1	1	1	1	7
Porcel(2007)	1	1	1	0	0	1	1	1	6
Zubairi(2007)	1	1	1	0	1	1	1	1	7
Yap(2008)	1	1	1	0	1	1	1	1	7
Karabulut(2008)	1	1	1	0	0	1	1	1	6
Lee(2010)	1	1	1	0	1	1	1	1	7
Pfeil(2010)	1	1	1	0	0	1	1	1	6
Sandevski(2012)	1	1	1	0	1	1	1	1	7
Comert(2013)	1	1	1	0	0	1	1	1	6
Rad(2014)	1	1	1	0	1	1	1	1	7
Liu(2015)	1	1	1	0	1	1	1	1	7
Panjwani(2019)	1	1	1	0	1	1	1	1	7
Chrysikos(2022)	1	1	1	0	1	1	1	1	7
Kumamaru(2016)	1	1	1	1	1	1	1	1	8
Choi(2017)	1	1	1	1	1	0	1	1	7
Kiris(2017)	1	1	1	1	1	1	1	1	8
Yildizeli(2018)	1	1	1	1	1	1	1	1	8
Levy(2020)	1	1	1	1	1	1	1	1	8
Buyuksirin(2021)	1	1	1	1	1	0	1	0	6
Zhang(2021)	1	1	1	1	1	1	1	1	8
